# Supplementary material for: Cross-species examination of X-chromosome inactivation highlights domains of escape from silencing
Source: Epigenetics Chromatin. 2021 Feb 17;14:12. doi: 10.1186/s13072-021-00386-8 (PMC7890635; doi:10.1186/s13072-021-00386-8)
Supplement: Supplementary file 1 — Additional file 1: Figure S1. The Xi/Xa expression ratio vs promoter DNAme level in individual human samples. Figure S2. The Xi/Xa expression ratio vs promoter DNAme level in individual mouse samples. Figure S3. Male vs female DNAme across species. Figure S4. A comparison of imprinted genes and genes subject to XCI. Figure S5. Comparison of DNAme data generated using WGBS and the 450 k array. Figure S6. Cross-species comparison of a primate-specific escape domain. Figure S7. Number of repeats within 15kb per TSS for genes subject or escaping XCI across species. Figure S8. Tests on mouse CTCF of our model trained on human CTCF. Figure S9. Mean female/male ATAC-seq signal across samples within 250 bp of TSSs, separated by tissue. Figure S10. Clustering of species by XCI status calls. [file 13072_2021_386_MOESM1_ESM.pdf]

Supplemental Tables

Table S1: All XCI status calls made in this study compared to humans.

Table S2: Individual XCI status calls per dataset. Each sheet is a separate dataset analyzed.

Table S3: The sources of data used in this study.

Table S4: Enrichment of repeats, CTCF and ATAC-seq at genes escaping vs subject to XCI. Repeats, CTCF and ATAC-seq are all on separate sheets. For repeats we tested the number of repeats within 15kb of each CpG island. For CTCF we tested the number of 200bp bins with predicted CTCF binding within 4kb of each TSS. For ATAC-seq we tested the female/male signal within 250bp of each TSS. We also included the number of CpG islands and TSSs per species that were informative for each analysis.

Table S5: **The number of predicted CTCF binding sites between genes in a discordant region.** A DanQ model was given overlapping 200bp bins of each genome and predicted the likelihood of it containing a CTCF binding site. The number of bins with over 80% chance of having CTCF binding were counted per region (A). Each region goes from either the start of a gene to its end, or from the end of one gene to the start of the next. Edges were included 5kb from the furthest gene on each side. This discordant region is the one featured in figure S6. The mean value region species for each region (B)

CEEHRC

Blueprint

	escape	subject	VE	no call
escape	43	0	7	8
subject	0	360	0	144
VE	1	1	6	10
no call	4	1	2	299

CEEHRC

CREST

	escape	subject	VE	no call
escape	31	0	1	3
subject	0	349	1	95
VE	2	0	4	10
no call	15	13	9	356

CREST

Blueprint

	escape	subject	VE	no call
escape	33	0	4	21
subject	1	424	0	79
VE	0	3	4	11
no call	1	18	8	282

	Blueprint	CEEHRC	CREST
N female	89	63	9
N male	64	39	12

Table S6: DNAme based XCI status calls compared across IHEC consortia. CREST, Blueprint and CEEHRC were the consortia with the most DNAme data sets when this data was downloaded. The majority of CEEHRC samples are cancer while the CREST samples and the majority of Blueprint samples are not. The 4th table shows the number of male and female samples per consortium. VE is variably escapes from XCI

Figure S1

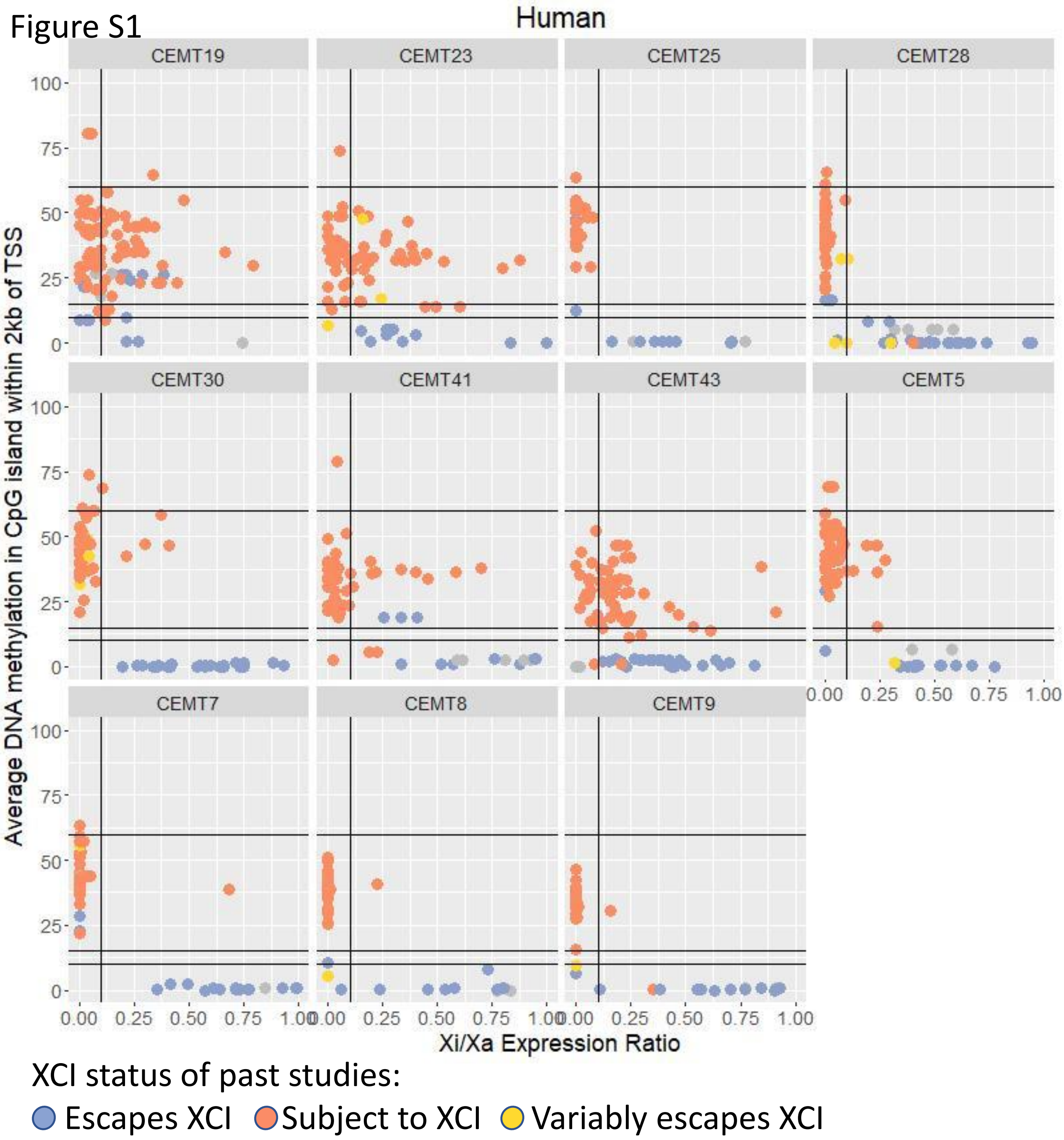

Figure S1: The Xi/Xa expression ratio vs promoter DNAme level in individual human samples. Each point is a SNP with Xi/Xa expression data, matched to the most likely promoter and any CpG islands within 2kb in order to have matched DNAme values. Lines are drawn at 0.1 Xi/Xa expression and at 10, 15 and 60% DNAme as they were used as thresholds to call XCI escape status later. Points are colored based on their XCI status calls in the previous literature (Balaton, 2015). CEMT30, a leukemia cancer sample, was used for Figure 1. Three samples (CEMT19, CEMT23 and CEMT43) were discarded from downstream analyses, because they did not appear to show skewing of Xi choice, with many genes called as subject to XCI by DNAme and previous studies, with an XiXa expression ratio >>0.1.

Figure S2

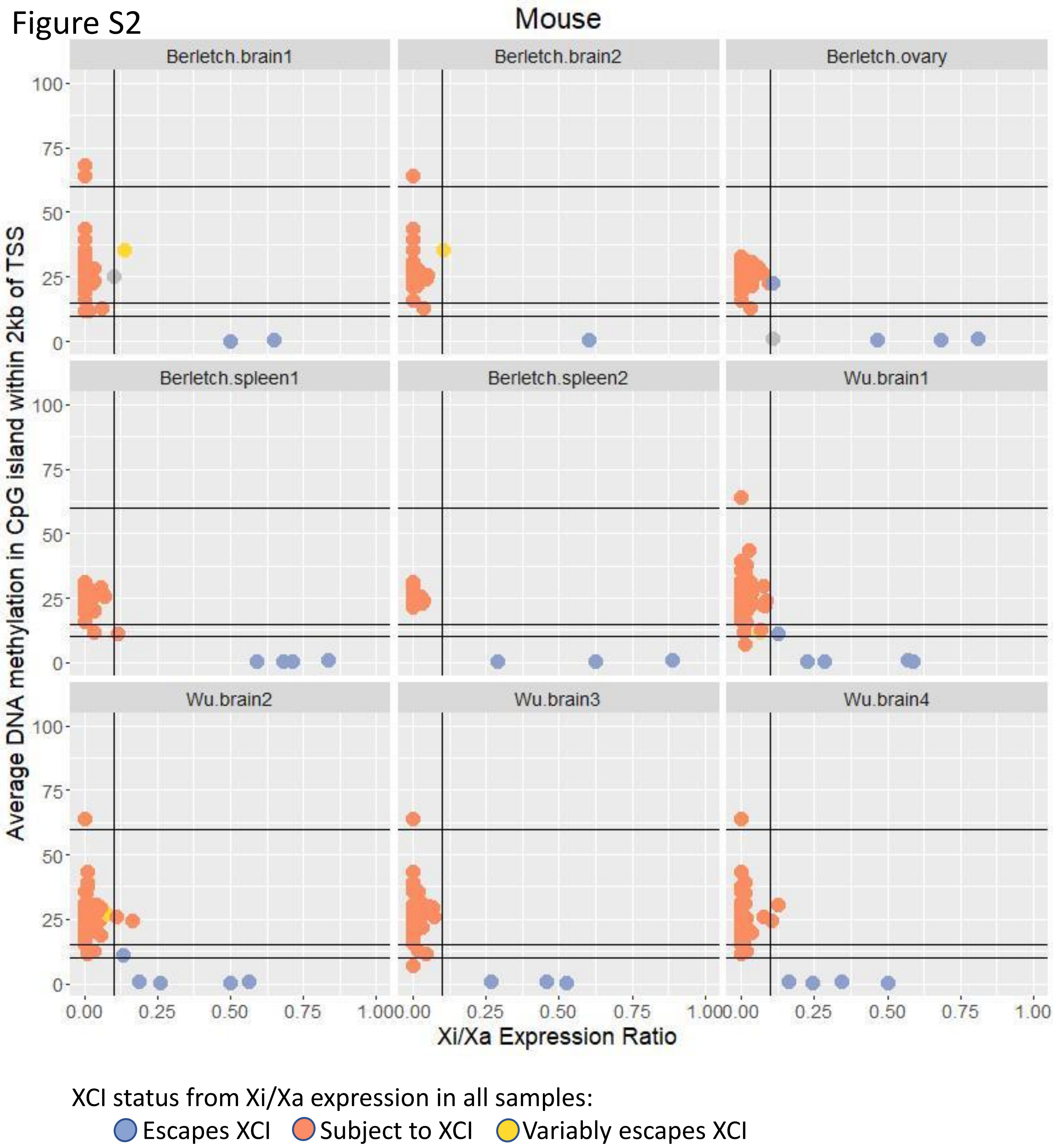

Figure S2: The Xi/Xa expression ratio vs promoter DNAme level in individual mouse samples. Each point is a SNP with Xi/Xa expression data, matched to the most likely promoter and any CpG islands within 2kb in order to have matched DNAme values. Lines are drawn at 0.1 Xi/Xa expression and at 10, 15 and 60% DNAme as they were used as thresholds to call XCI escape status later. Points are colored based on their XCI status calls made using Xi/Xa expression. Data from 2 different studies are used: one used an *Xist* knockout to skew Xi choice and the other used differently colored fluorescent proteins expressed from each X chromosome to sort cells based on Xi choice. Data from Keown, *et al*. not shown here was used for Figure 1.

Fig S3. **Male vs Female Methylation shows similarities across species**

Not pictured chimp (WGBS) and goat, due to lack of male data

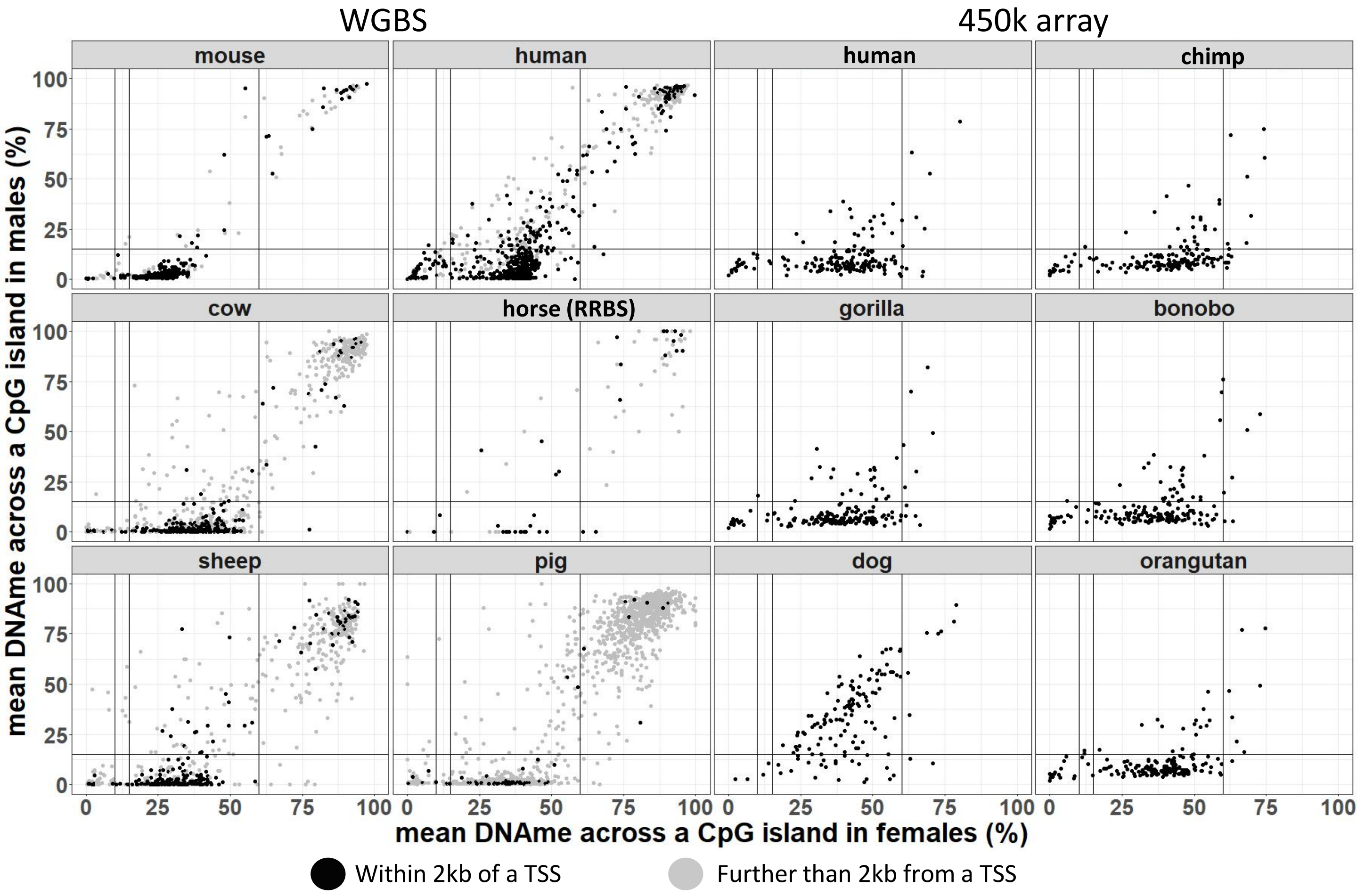

Figure S3: Male vs female DNAme across species. The DNAme data shown was generated with 3 different methods: WGBS, RRBS and the human 450k DNAme array. Each point is a CpG island. Lines are drawn at female DNAme of 10,15 and 60 as those thresholds were used to call a gene's XCI status and at male DNAme of 15 as genes with higher than 15% male DNAme were discarded from further analysis. CpG islands are colored based on the distance to their closest TSS.

Fig S4

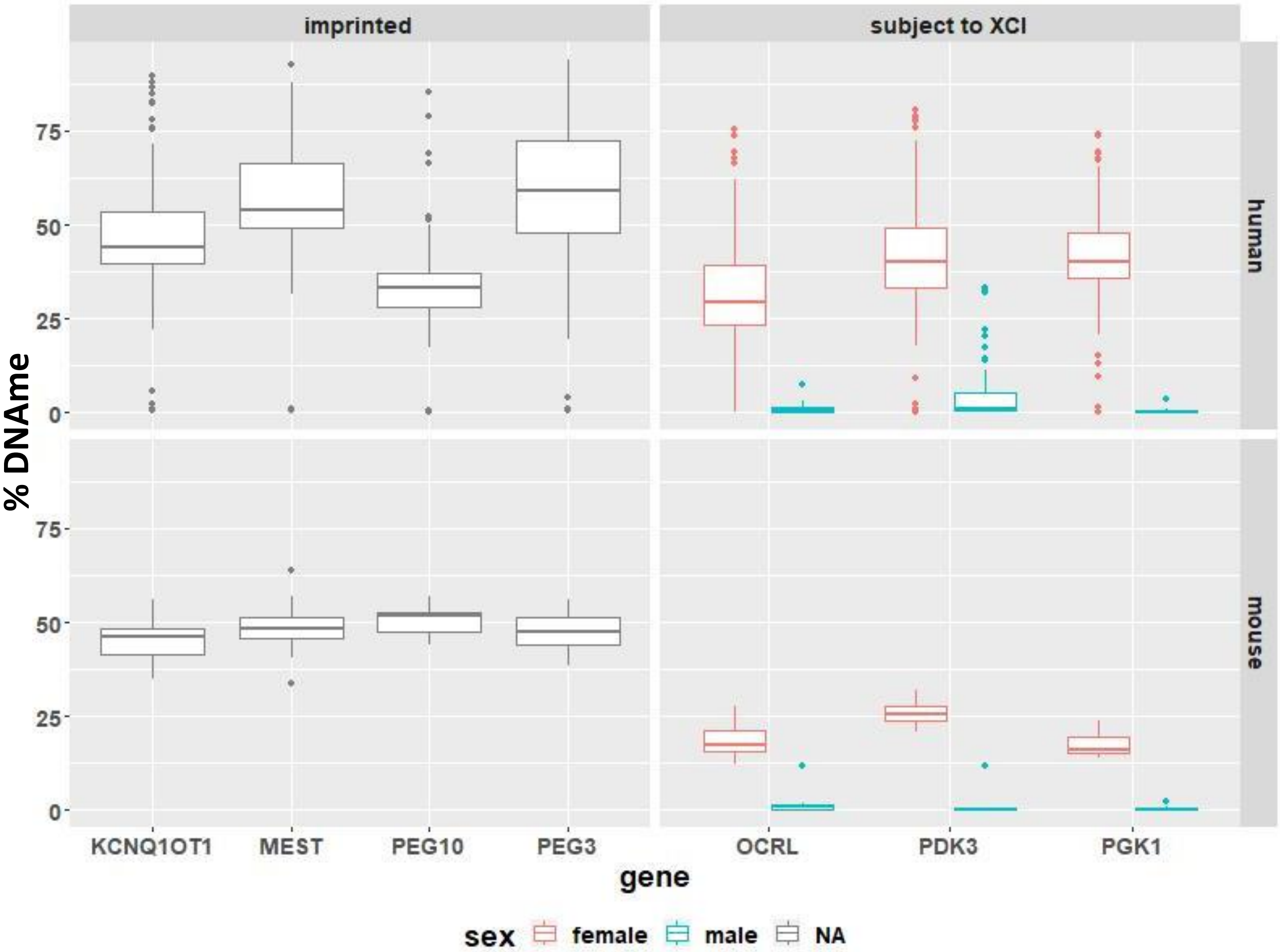

Figure S4: A comparison of imprinted genes and genes subject to XCI. The average DNAme level at promoter CpG islands are shown for 4 imprinted genes and 4 genes subject to XCI in humans (A) and mouse (B). Genes subject to XCI have males and females separate as females are expected to be hemi-methylated while males are expected to have low methylation.

Fig S5

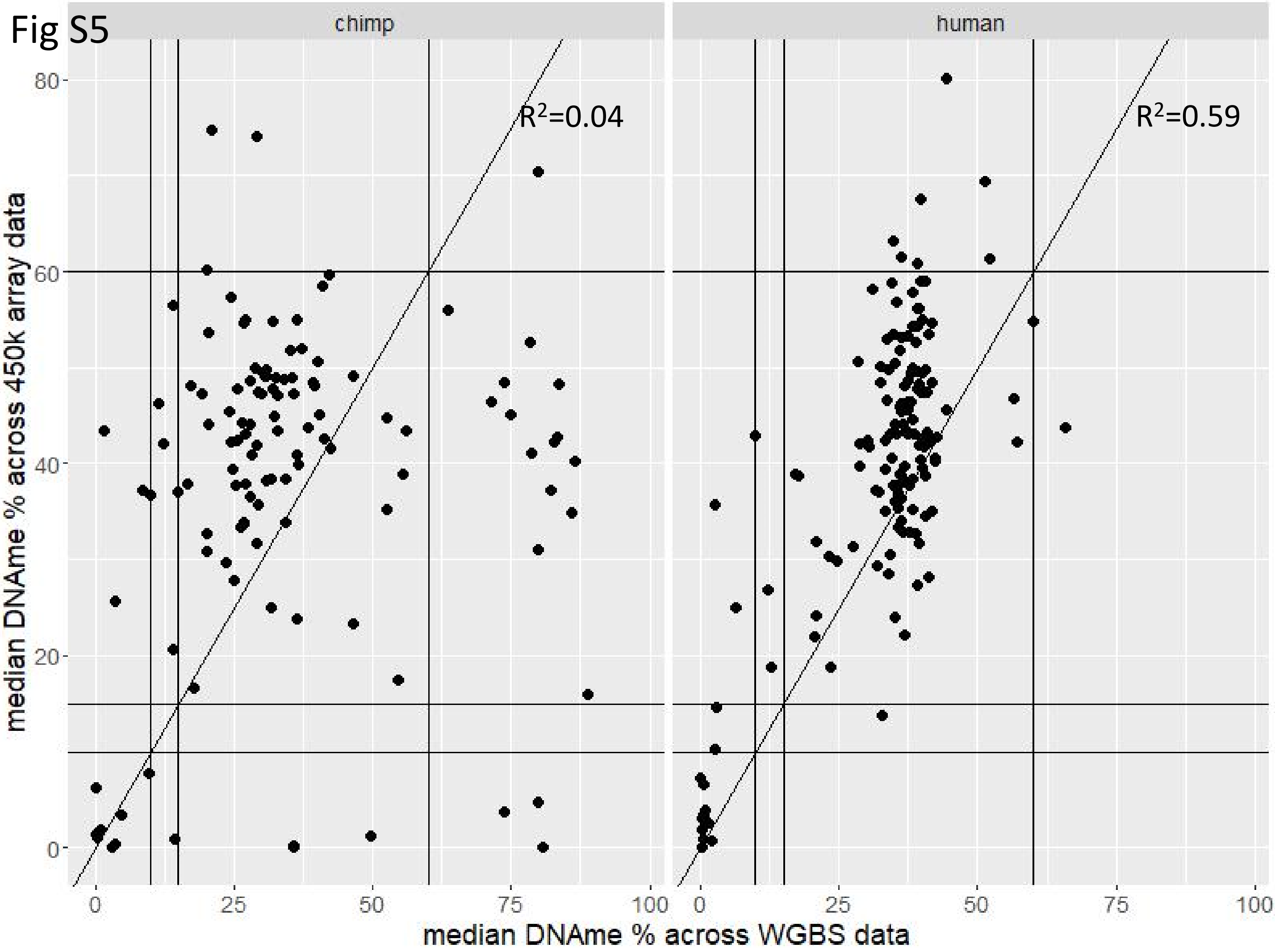

Figure S5: Comparison of DNAme data generated using WGBS and the 450k array. Human and chimp were the only two species which had data generated using both methods. Lines are drawn at 10,15 and 60% DNAme to show the thresholds used for calling XCI status. Another line was drawn along the diagonal to show where perfect concordance between datasets would be. The R^2 value was calculated showing the level of concordance found between the 2 methods.

Fig S6

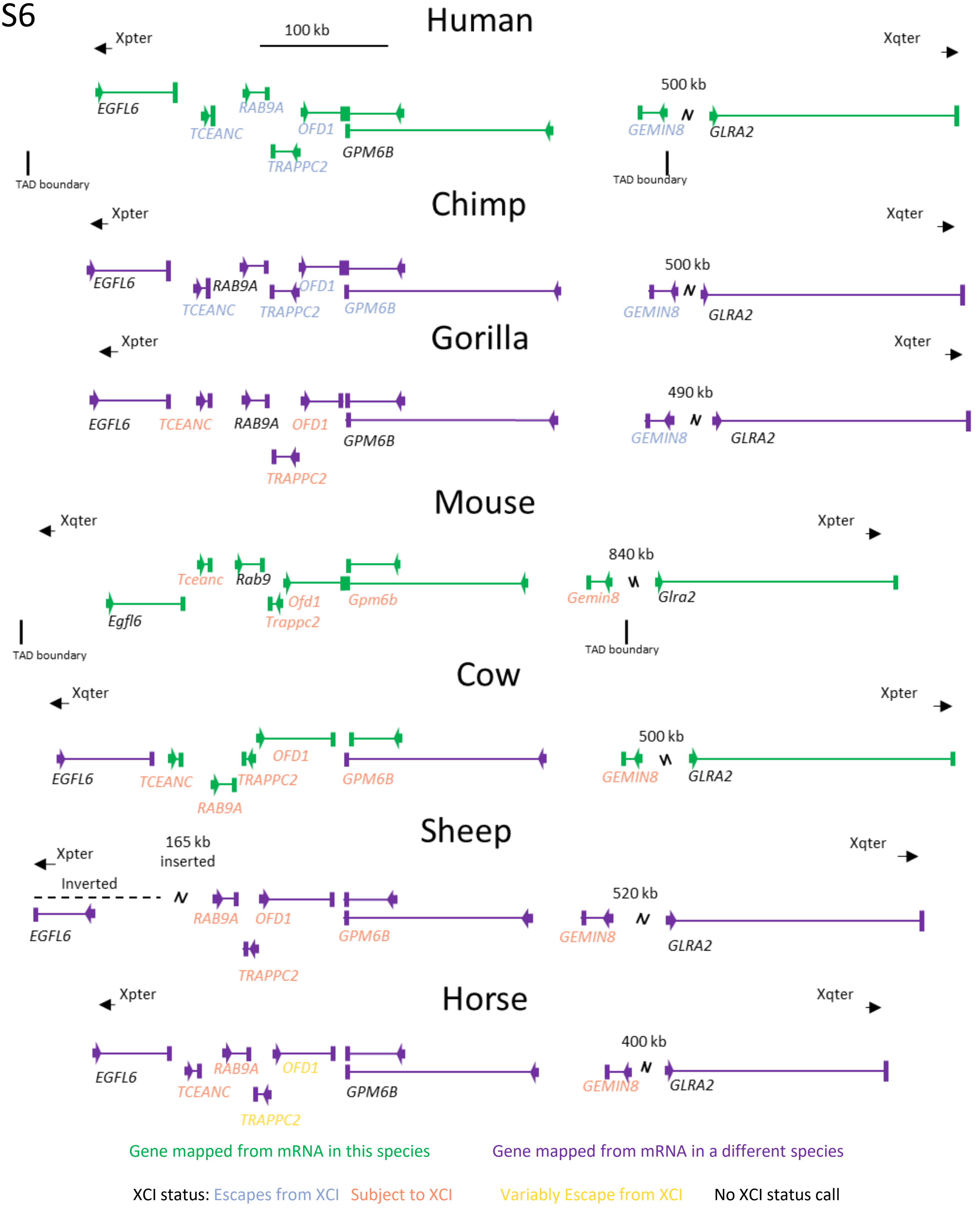

Figure S6: Cross-species comparison of a primate-specific escape domain. The domain spanning from *TCEANC* to *GEMIN8* and the neighboring gene on each side are shown. Genes names are colored by their XCI status in each species and the gene diagram is colored by whether the gene annotation is from mRNA in that species or from other species. All regions in all species were scaled together, with species aligned at the end of *GPM6B*. As there is a large gene-free region between *GEMIN8* and *GLRA2* this region has been condensed and the distance between the two genes noted. Dotted lines show the region that is inverted in sheep. Xpter and Xqter show the direction to the short and long arms of the chromosome respectively, note that this region and much of the X chromosome is inverted in mouse and cow [S1, S2, S3]. Cow had inconsistencies between bosTau6 (used in our data source and this study) and bosTau9 (the latest cow genome build), with bosTau6 being used here. bosTau9 had duplication or rearrangement of *EGFL6 and TCEANC*. Gorilla and horse had small pseudo-gene insertions in the region, but these were only around 2kb in size and so were left out.

Fig S7

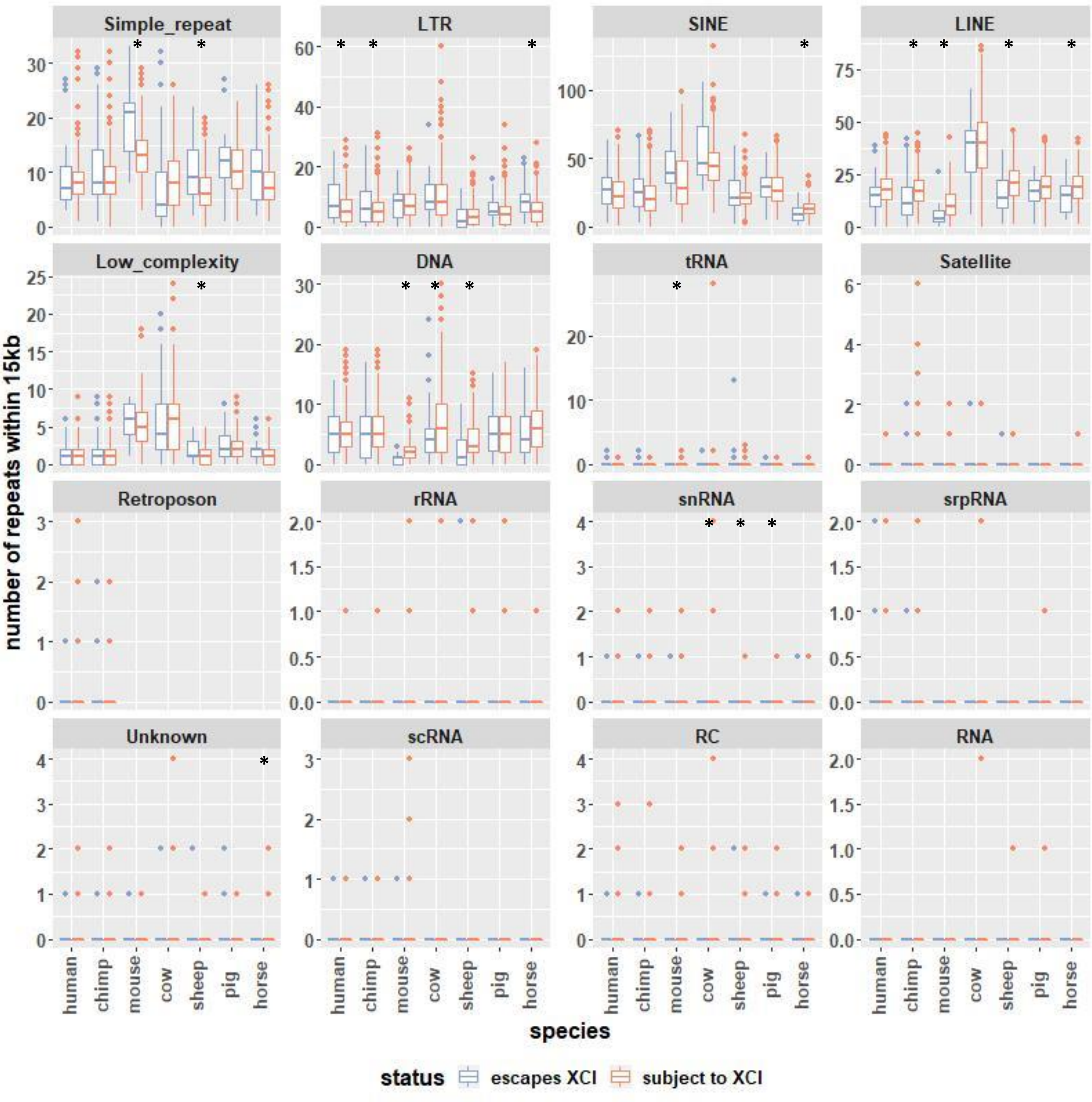

Figure S7: Number of repeats within 15kb per TSS. Species with a * have significant differences between genes found escaping XCI and those found subject to XCI at adjusted p-value<0.01.

Fig S8

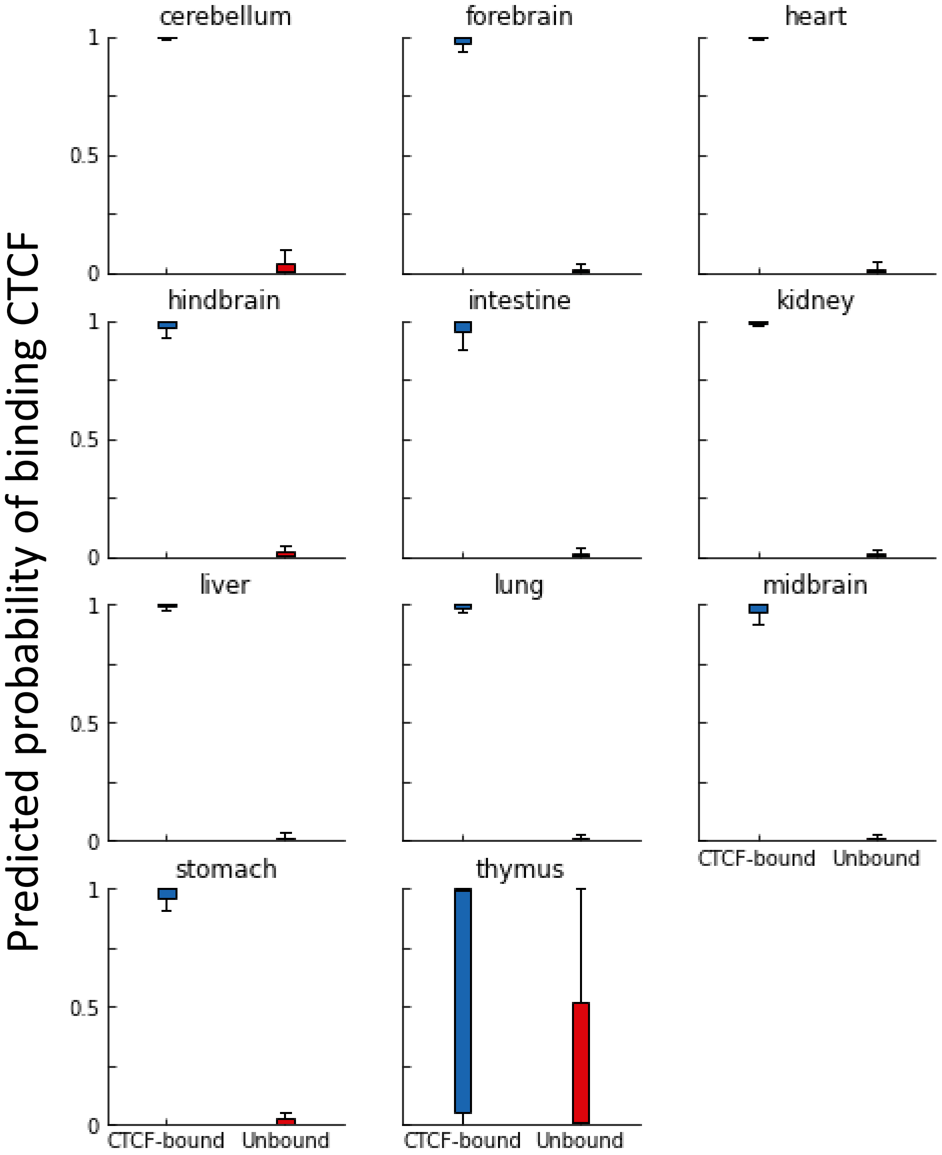

Figure S8: Tests on mouse CTCF of our model trained on human CTCF. This is a DanQ model trained on human CTCF ChIP data from ENCODE and tested on mouse data from ENCODE.

Fig S9

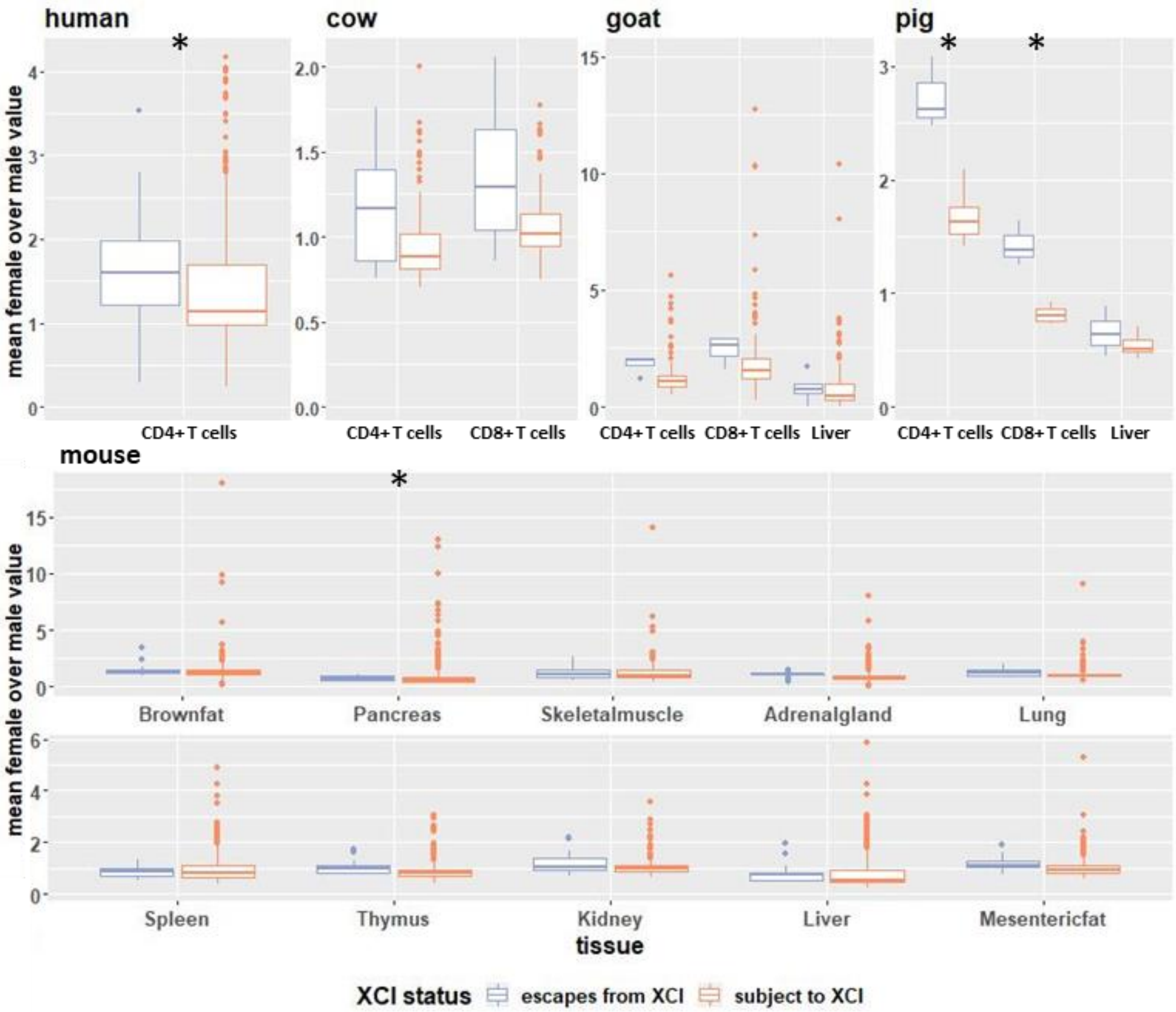

Figure S9: **Mean female/male ATAC-seq signal across samples within 250bp of TSSs, separated by tissue.** Tissues with a ***** have significant differences between genes found escaping XCI and those found subject to XCI at adjusted p-value<0.01.

Fig S10

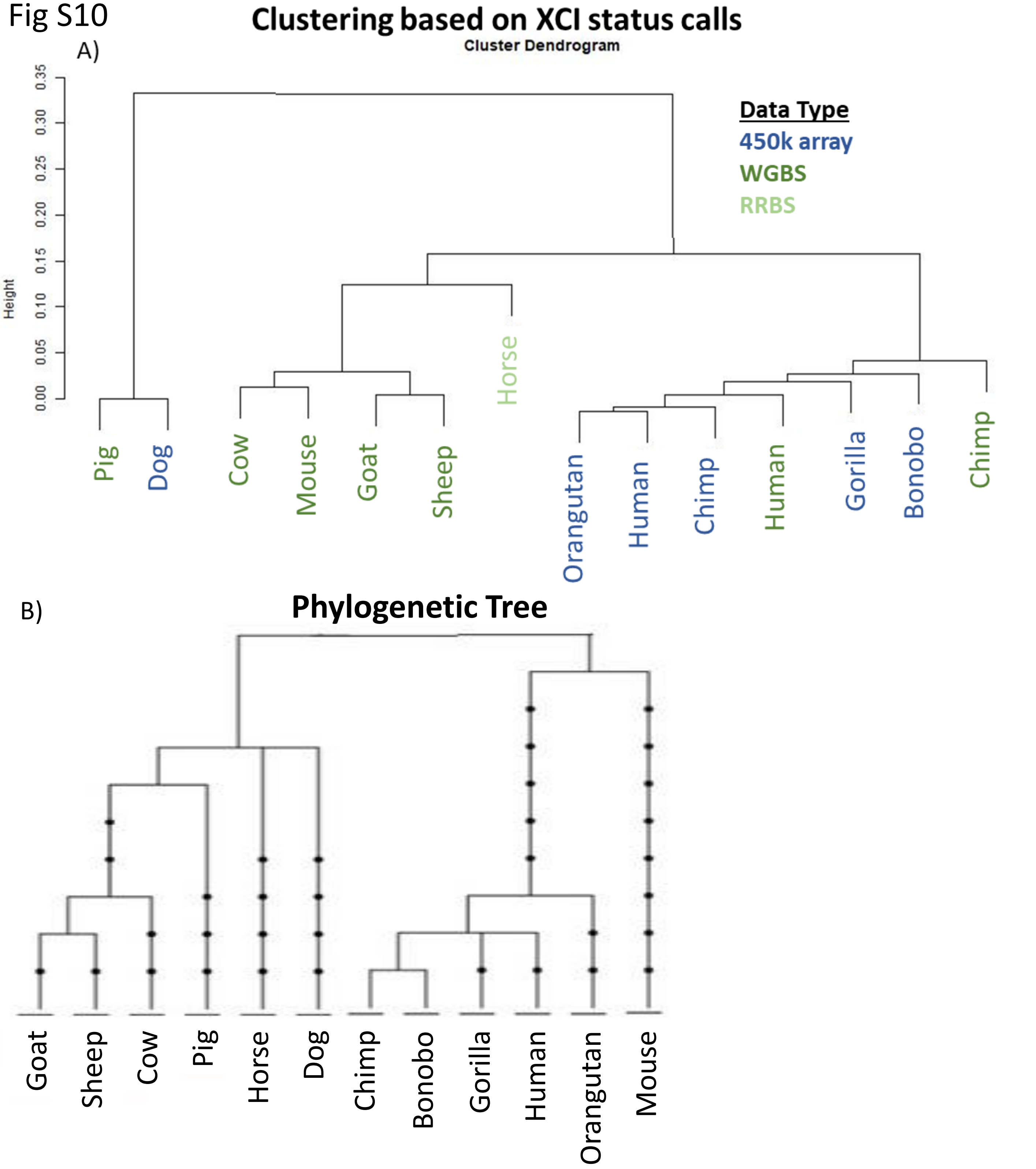

Figure S10: **Clustering of species by XCI status calls.** Species were clustered by their XCI status calls (A) and compared to a phylogenetic tree showing their evolutionary relations (B). For the clustering, species names are colored by the type of data used to generate the XCI status calls.

Supplemental References

S1 Balaton BP, Brown CJ. Escape Artists of the X Chromosome. Trends Genet. 2016;32:348–59.

S2 Proskuryakova AA, Kulemzina AI, Perelman PL, Makunin AI, Larkin DM, Farré M, et al. X Chromosome Evolution in Cetartiodactyla. Genes. 2017;8. Available from: http://dx.doi.org/10.3390/genes8090216

S3 Sandstedt SA, Tucker PK. Evolutionary strata on the mouse X chromosome correspond to strata on the human X chromosome. Genome Res. 2004;14:267–72.
