# Supplementary material for: Cross-species examination of X-chromosome inactivation highlights domains of escape from silencing
Source: Epigenetics Chromatin. 2021 Feb 17;14:12. doi: 10.1186/s13072-021-00386-8 (PMC7890635; doi:10.1186/s13072-021-00386-8)
Supplement: Supplementary file 2 [file 13072_2021_386_MOESM7_ESM.docx]

CEEHRC

Blueprint	escape	subject	VE	no call
**escape**	43	0	7	8
**subject**	0	360	0	144
**VE**	1	1	6	10
**no call**	4	1	2	299

CEEHRC

CREST	escape	subject	VE	no call
**escape**	31	0	1	3
**subject**	0	349	1	95
**VE**	2	0	4	10
**no call**	15	13	9	356

CREST

Blueprint	escape	subject	VE	no call
**escape**	33	0	4	21
**subject**	1	424	0	79
**VE**	0	3	4	11
**no call**	1	18	8	282

	Blueprint	CEEHRC	CREST
**N female**	89	63	9
**N male**	64	39	12

Table S6: DNAme based XCI status calls compared across IHEC consortia. CREST, Blueprint and CEEHRC were the consortia with the most DNAme data sets when this data was downloaded. The majority of CEEHRC samples are cancer while the CREST samples and the majority of Blueprint samples are not. The 4th table shows the number of male and female samples per consortium. VE is variably escapes from XCI
